# Characterization of Some Stilbenoids Extracted from Two Cultivars of Lambrusco—*Vitis vinifera* Species: An Opportunity to Valorize Pruning Canes for a More Sustainable Viticulture

**DOI:** 10.3390/molecules28104074

**Published:** 2023-05-13

**Authors:** Veronica D’Eusanio, Francesco Genua, Andrea Marchetti, Lorenzo Morelli, Lorenzo Tassi

**Affiliations:** 1 Department of Chemical and Geological Sciences, University of Modena and Reggio Emilia, 41121 Modena, Italy; f.genua9@gmail.com (F.G.); andrea.marchetti@unimore.it (A.M.); lollo2200@gmail.com (L.M.); 2 National Interuniversity Consortium of Materials Science and Technology (INSTM), 50121 Florence, Italy; 3 Interdepartmental Research Center BIOGEST-SITEIA, University of Modena and Reggio Emilia, 42121 Reggio Emilia, Italy

**Keywords:** sustainability, stilbenoids, roasted grape pruning canes, *Vitis vinifera*, Lambrusco

## Abstract

Pruning canes from grape vines are valuable byproducts that contain resveratrol and other health-boosting stilbenoids. This study aimed to assess the effect of roasting temperature on the stilbenoid content of vine canes by comparing two *Vitis vinifera* cultivars, Lambrusco Ancellotta and Salamino. Samples were collected during different phases of the vine plant cycle. One set was collected in September after the grape harvest and was air-dried and analyzed. A second set was obtained during vine pruning in February and evaluated immediately after collection. The main stilbenoid identified in each sample was resveratrol (~100–2500 mg/kg), with significant levels of viniferin (~100–600 mg/kg) and piceatannol (~0–400 mg/kg). Their contents decreased with increasing roasting temperature and residence time on the plant. This study provides valuable insights into the use of vine canes in a novel and efficient manner, which could potentially benefit different industries. One potential use involves the roasted cane chips to accelerate the aging of vinegars and alcoholic beverages. This method is more efficient and cost-effective than traditional aging, which is slow and unfavorable from an industrial perspective. Furthermore, incorporating vine canes into maturation processes reduces viticulture waste and enhances the final products with health-promoting molecules, such as resveratrol.

## 1. Introduction

Among the world’s fruit crops, viticulture is certainly one of the most important, with a grape production of about 73 million tons in 2021 [1]. Its economic role is crucial, particularly in winemaking [2], which is one of the most powerful production sectors in different regions of the world [3]. Italy, France, and Spain together account for 51% of total wine production [1], making the wine sector a benchmark for the EU’s primary production areas [2]. Grape cultivation and vinification result in significant amounts of waste and by-products, including pomace, seeds, stems, pruning canes, yeast and bacterial lees, organic acids (i.e., tartrate), CO_2_, and water. Therefore, the viticulture sector has significant socio-economic potential, as agro-industrial waste can be valorized to obtain high-value products [4]. To improve the efficiency and environmental impact of the wine industry, it is necessary to identify strategies to minimize waste production and effectively recover it from a circular economy perspective [5]. The recovery of bioactive molecules through biorefinery processes, such as polyphenols and stilbenes, proteins, edible and essential oils, dietary fibers, pectins, and simple sugars, aligns with the Agenda 2030 for Sustainable Development Goals (SDGs) [6]. In particular, SDG 12 aims to significantly reduce waste production through prevention, reduction, recycling, and reuse, suggesting a new growth paradigm that supports the preservation of natural resources and the environment, social welfare, and economic prosperity [7,8]. It follows that the implementation of circular economy principles increases the added value of winemaking by-products, thereby reducing the environmental impact of the entire production sector. In addition, biorefinery residues can be destined for waste-to-energy plants to complete the entire cycle, thus maximizing profits for operators and stakeholders.

Consumers are increasingly looking for healthy and functional foods enriched with bioactive molecules that possess specific health properties such as antioxidant, anti-aging, and anti-inflammatory properties. Several studies have shown that agri-food waste is a valuable source of these analytes [4,5,9,10] and has enormous potential to meet the growing market demand. Stilbenes, found in viticulture by-products, are a particularly interesting class of antioxidants, as they have been associated with a reduced risk of many diseases [11], including cardiovascular diseases [12] and cancer [13]. In particular, *trans*-resveratrol has gained worldwide attention for its beneficial effects on human health. In addition, its oxidative polymerization produces oligomers, including *trans*-ε-viniferin, which have many beneficial biological properties [14,15,16], including antimicrobial, anticancer, hepatoprotective, and antioxidant properties [17,18]. These compounds are found in grapes, berries, and nuts but are also readily contained in viticulture by-products. The extraction of stilbenoids from grapevine pruning canes has been proposed as a cost-effective method for obtaining high-value phytochemicals [11,19,20,21]. They represent a large amount of waste from grape cultivation, with an estimated volume of 1–5 tons per hectare, depending on various factors, such as planting density, climate, and vigor of the grape cultivar [22].

A possible application of vinewood is the use of shavings or tannic extract to enhance the flavor of balsamic vinegar [23], a traditional product from the Modena and Reggio Emilia district [24]. The latter is produced with cooked grape must, through a maturation cycle, lasting several years, leaving the product in contact with different wood barrels (i.e., mulberry, acacia, oak, cherry, chestnut, etc.) [25]. As the wood slowly diffuses, it enriches vinegar with aromatic molecules and improves its quality and sensory characteristics [26,27,28]. The presence of woody molecules increases the complexity of aromatic bouquets, particularly sought after in aged products with high commercial value. However, the slow aging process in barrels over long periods significantly limits the operation of the production chain [29]. This, together with other factors, such as the high price of the barrels, their limited duration, the large storage space required, and the required maintenance, makes traditional maturation systems expensive and laborious. Similarly, the aging of wines or other alcoholic products, such as brandy and whiskey, is carried out in this manner [24]. Therefore, simpler and more cost-effective aging systems have been developed as alternatives to the traditional methods [26]. The use of wood shavings immersed in maturing vinegar accelerates ripening and shortens contact time without reducing the quality of the food product.

Another alternative involves the direct addition of tannic extracts obtained from selected woods of interest. Both methods yield sensorial properties similar to those of the product naturally aged in barrels, maintaining the same woody essence derived from either the shavings or the wood that generated the extract. The addition of tannic extracts is a more practical and adaptable solution, as it allows for an accurate dosage of ingredients, ensuring consistent organoleptic features for different production batches over time. Many factors affect the aromatic profiles of agronomic products produced using alternative aging systems. The amount of volatile organic compounds (VOCs) and non-volatile compounds generated by shavings and released by contact, or introduced as tannic extracts into the product, depends on the particular characteristics of the wood used (botanical and geographical origin, seasoning, genotype), contact surface (size, amount of shavings), the duration of solid–liquid direct contact, and, above all, the degree of roasting. In fact, wood roasting imparts more marked and refined aromatic notes; therefore, it is widely used to produce vinegar aging barrels.

In this study, pruning canes of two Lambrusco wine cultivars characteristic of the Emilia-Romagna region (Italy), Ancellotta and Salamino, were examined. The shavings were thermally treated at 180 °C, 200 °C, 220 °C, and 240 °C, and the contents of *trans*-resveratrol, *trans*-ε-viniferin, and other resveratrol oligomers in their ethanolic extracts were investigated using HPLC-MS. To better understand vine cane utilization, we carried out two rounds of pruning, collecting one set of samples after grape harvesting in September and air-drying them in the laboratory, while gathering another set during February vineyard pruning and analyzing them immediately thereafter. In addition, the headspace compositions of two samples roasted at 200 °C were evaluated using HS-SPME-GC-MS. The goal was to determine the main volatile aromas formed as a result of heat treatment.

The main aim of our study was to examine the influence of sampling time and roasting temperature on the stilbenoid content in vine canes, with the aim of determining effective strategies for extracting valuable products from these residues.

## 2. Results and Discussion

### 2.1. Proximate Analysis

The chemical composition and physical properties of vegetable matrices are significantly influenced by certain factors, including geographical origin, degree of ripeness, and the cultivar to which they belong [4,10]. Table 1 lists the proximate chemical composition of the samples.

For all the samples examined, it was generally observed that the moisture content of the PH samples was approximately 9% higher than that of the PP samples, likely due to differences in their natural drying processes. The PH samples were only partially dried naturally and analyzed immediately after collection. Conversely, the PP samples underwent a more extensive natural drying process, as they were collected a long time after grape harvesting (approximately after six months). The Ancellotta cultivar has lower moisture levels than Salamino, which could be linked to various genotypic factors, such as greater stiffness and compactness, along with the lower porosity of the wood. These attributes can also be influenced by external factors, such as the plant’s maturity level, the amount of sunlight it received, its location, and soil type.

In the production of vinegars and alcoholic beverages, the wood porosity of the barrel is a crucial factor in determining the fermentation process. If the porosity is higher, there will be a greater oxidation of polyphenolic compounds, as more oxygen will be exchanged during aging [25,30]. However, using infused wood shavings can reduce these differences in porosity and minimize their effects on the fermentation process [25].

### 2.2. Effect of the Roasting Temperature on the Samples’ Characteristics

To obtain a comprehensive understanding of the changes that roasting temperature induces in the composition of matrices, it is important and indispensable to evaluate mass losses. Although they appear to be basic data, they enable a quick assessment of the degree of change resulting from the heat treatment. In addition, comparing data from different samples can help draw conclusions regarding differences in composition between the two cultivars under examination, particularly with regard to their heat resistance. We also anticipate that the timing of cane sample collection (post-harvest or post-pruning) will significantly impact this dataset as the composition differs greatly.

**Figure 1:**
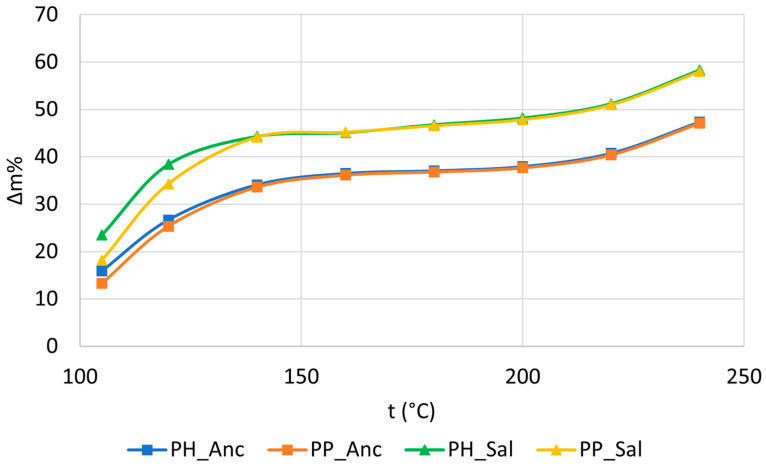
Trend of mass loss values following roasting of the grapevine samples.

The mass loss results are listed in Table 2 and graphically displayed in Figure 1.

Table 2 shows that the grapevine canes of the two cultivars behaved differently when exposed to roasting temperature. The aromatic profile of toasted wood varies progressively with roasting temperature because the composition of the gas phase produced varies [31]. This aspect is further explored in Section 2.4; however, the mass loss provides us with the preliminary information necessary to understand which of the samples emit the greatest number of VOCs following roasting.

The Salamino cultivar lost over 10% of its mass at each thermal stage when compared to the Ancellotta variety, indicating that the latter has more compact and heat-resistant wood. After the removal of residual moisture at 105 °C, together with the high vapor pressure VOCs responsible for the typical woody aroma, a slow increase in mass loss by elimination of semi-volatile organic compounds (SVOCs) was recorded up to ~140 °C. This trend is clearly visible in Figure 1, which shows the drying-toasting treatment of the four samples compared. Furthermore, observing the initial stretch of the curves, it can be hypothesized that the Salamino cultivar undergoes more post-harvest water stress than the Ancellotta cultivar. Subsequently, the “water-VOCs-SVOCs” gap between PH and PP samples was minimized when the pruning shavings were heated to ~140 °C, and the curves relating to each variety tended to overlap. The effects of roasting became significant at approximately 160 °C as the wood gradually stiffened, losing the typical characteristics of elasticity. At this temperature, the removal of structural water begins [32], a process that persists throughout the entire thermal range. Proteins persist up to ~200–220 °C [33,34], suggesting that the chemical structure of the biomass begins to destabilize and partly depolymerize. The structural decay reactions of proteins occur up to 240 °C, leading to strong mass loss from 220 °C to 240 °C. Moreover, above 200 °C, cellulose and hemicellulose begin to gradually decompose [31,35,36]. Lastly, the higher initial moisture of the PH samples was responsible for their greater mass loss compared to that of the PP samples.

The extraction yield (Table 3) is a further parameter that allows us to understand the compositional differences between the different matrices examined in a simple way. A higher extraction yield is associated with a greater quantity of extracted polyphenols, tannins, antioxidants, and mineral salts, among other components.

**Figure 2:**
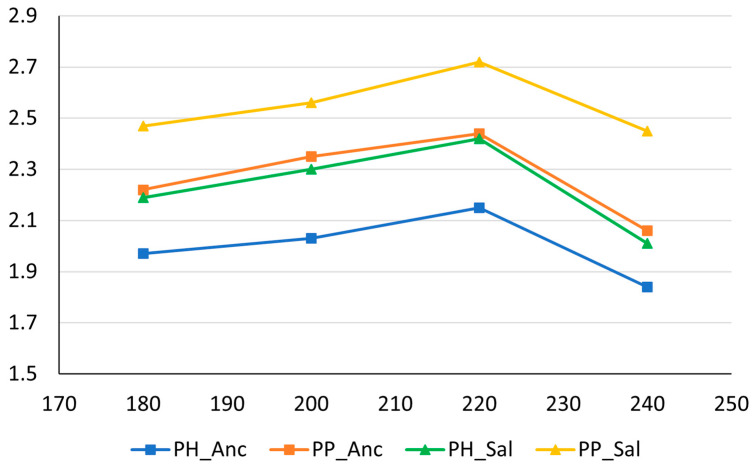
Trend of the extraction yield values with respect to roasting temperature.

Figure 2 shows the trend of the extraction yield values with respect to roasting temperature.

The results showed a consistent trend for both the cultivars examined, regardless of the source of the PH or PP samples. The yield increased gradually as the roasting temperature increased to 220 °C, reaching a maximum for all the samples. A sudden decline in the yield was observed at 240 °C. It is important to highlight that the samples treated at 240 °C were extensively toasted and dark black in color. This was accompanied by a considerable mass loss, and the resulting aromatic components had a wood tar-like aroma, which is probably classifiable as off-flavors. Moreover, the extraction yield of the PP samples was 11–12% higher than that of the PH samples. This evidence is in line with previous studies [37], which suggest that even 30–40 days after pruning, aged vine wood continues to synthesize analytes and stilbenoids through enzymatic reactions. However, it should be noted that the thermal degradation and pyrolytic processes play a significant role in increasing the extraction yield as well, triggering the formation of off-flavor compounds.

### 2.3. Stilbenoids Concentration in the Different Samples of Roasted Grape Pruning Canes

**Figure 3:**
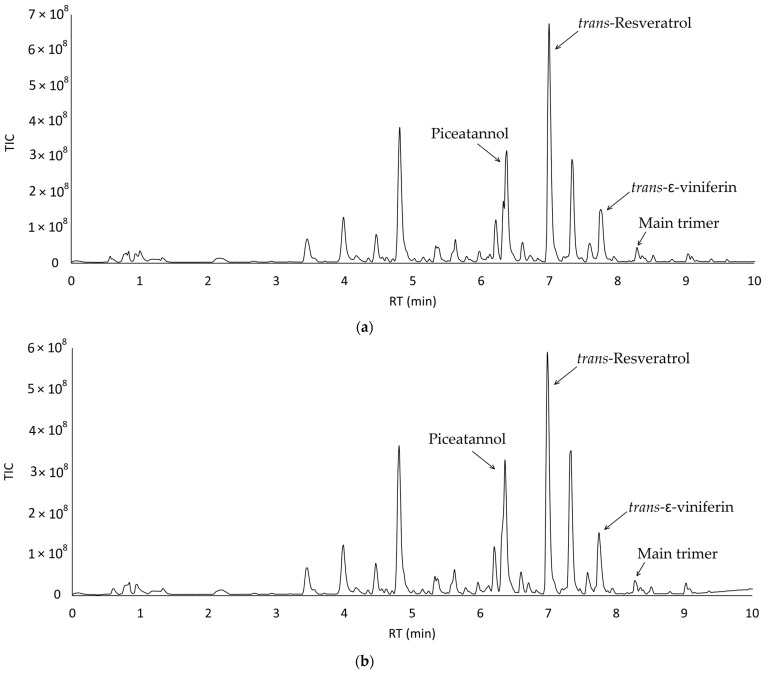
(**a**) UHPLC-MS/MS chromatogram of the ethanolic extracts of the sample PP_Anc200; (**b**) UHPLC-MS/MS chromatogram of the ethanolic extracts of the sample PP_Sal200.

UHPLC-MS/MS is a widely recognized technique for the accurate detection of stilbenes [38,39]. As an example, Figure 3a,b show two representative chromatograms of the reversed-phase UHPLC-MS analysis of the ethanolic extracts of PP_Anc200 (Figure 3a) and PP_Sal200 (Figure 3b).

**Figure 4:**
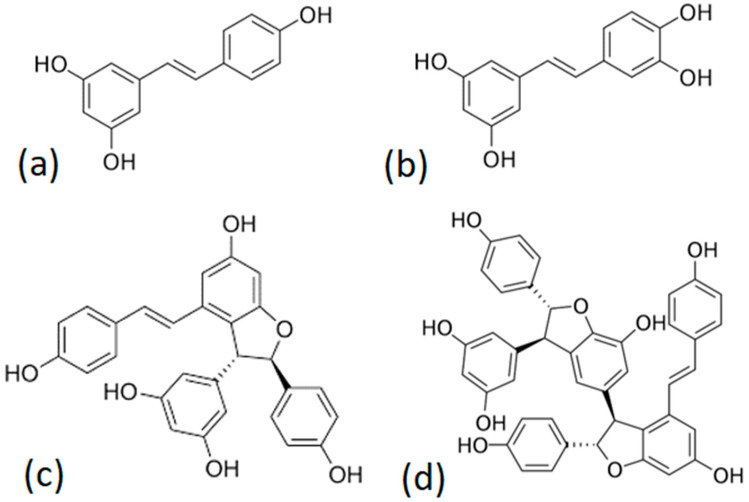
Structures of the identified stilbenoids: *trans*-resveratrol (**a**): *trans*-piceatannol (**b**); *trans*-ε-viniferin (**c**); myabneol C (**d**).

Stilbenoid quantification involved the measurement of only four critical compounds (i.e., *trans*-resveratrol, *trans*-ε-viniferin, *trans*-piceatannol, and the main trimer of *trans*-resveratrol) [39]. The structures of the identified stilbenoids are shown in Figure 4. We reported the structure of myabenol C (Figure 4d) as the main trimer, as it has been widely reported in the literature as the main trimer of *trans*-resveratrol in vine canes.

The remaining stilbenoids were below the quantification limit of the implemented methodology, which is in line with the results of previous studies by Vergara et al. [40]. The total stilbenoid concentration was calculated as the sum of the concentrations of the four major stilbenes, quantified as *trans*-resveratrol equivalents. The main trimer, with a molecular ion [M–H]^−^ of 679 amu, probably corresponds to one of the isomers of miyabenol C [40,41].

Several studies have demonstrated that *trans*-resveratrol exhibits a multitude of important biological and pharmacological functions [42,43,44], such as protecting against metabolic and cardiac diseases, and has anti-aging, anticarcinogenic [13,45], and inti inflammatory properties [46]. Additionally, it has been shown to enhance the longevity of many species and is thought to replicate the effects of calorie restriction [47,48,49]. The less-studied trans-piceatannols also displayed many biological activities, including chemopreventive and antioxidant properties [50,51]. It has also been studied for its potential in treating metabolic diseases, such as type 2 diabetes, as it is thought to have an insulin-like effect on the body. Furthermore, bioassays testing oligomers and analogues of *trans*-resveratrol revealed their board pharmacological potential [14,15,16,52,53]. These compounds are phytoalexins, which are natural plant compounds produced in response to stresses, such as pathogen attack, UV radiation, and mechanical damage [54].

Table 4 presents the concentrations of the major stilbenoids in the samples roasted at different temperatures.

For the same sample collection time and pre-treatment temperature, the stilbenoid concentration values for the Salamino cultivar were lower than those of Ancellotta. Stilbenoid content changes greatly with the cultivar, as genotypic factors greatly affect their bioaccumulation [19,55]. On average, *trans*-resveratrol had the highest values, followed by *trans*-ε-viniferin, *trans*-piceatannol, and the main trimer of *trans*-resveratrol. Table 4 shows a decrease in the concentration values as the roasting temperature increases, and this behavior is not disregarded due to the strong thermal instability of stilbenoids. This leads to the complete non-quantification of the main trimer in the samples roasted at 240 °C. The transition from 220 °C to 240 °C, in fact, involves considerable stress on the matrix, as confirmed by the high mass loss that occurred, as shown in Table 2 (Section 2.2).

An important aspect concerns the collection time for pruning cane samples. In particular, the PP samples, collected after grapevine pruning and partially dried at room temperature, had significantly higher concentrations. Previous studies have shown that, at the time of collection, pruning cane samples contain high levels of oligomeric stilbenoids, but almost no monomeric stilbenoids, such as *trans*-resveratrol and -piceatannol [22,37]. These accumulate at various rates during storage because the drying process is perceived as a stress signal by living tissues. Monomeric stilbenoid biosynthesis is induced in response to biotic and abiotic stress [56,57,58]. The concentration range of *trans*-resveratrol for the PP samples was approximately 600–2500 mg kg^−1^, whereas that for the PH samples was 100–900 mg kg^−1^. According to the available literature [19,40], the concentration of *trans*-resveratrol in thermally untreated samples varies between 400 and 7000 mg kg^−1^, with a strong dependence on the grape cultivar. Despite being heat treated, the PP samples still produced values that fell within this range.

**Figure 5:**
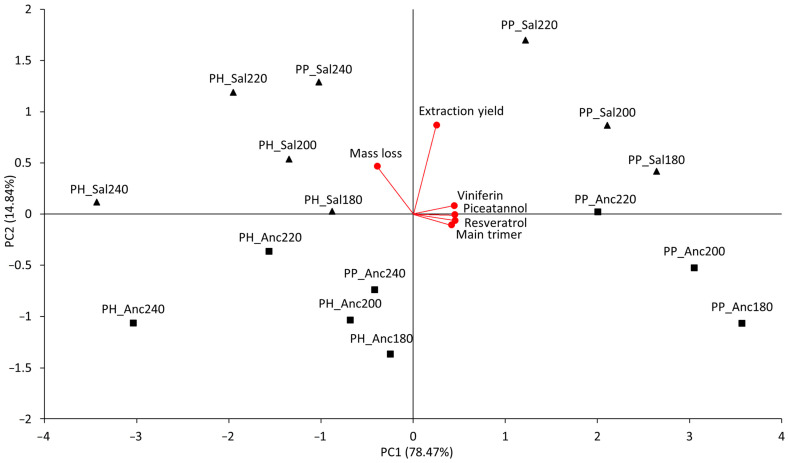
PCA biplot of the total stilbenoids content for the 16 grape shives samples (explained variance reported in brackets).

The principal component analysis (PCA) of the mean content of the four quantified stilbenoids in the 16 samples (Figure 5) revealed that 93.31% of the total variance was explained by PC1 and PC2. It is evident that: (i) PH samples contain fewer stilbenoids than PP samples, particularly PH_Sal240 and PH_Anc220; (ii) increasing the roasting temperature of cane pruning shives reduces stilbenoid content; (iii) the higher the roasting temperature, the more the mass loss becomes important for differentiating the samples under examination; and (iv) the extraction yield mainly differentiates the PP samples roasted at 220 and 240 °C.

Finally, it is important to consider the potential presence of pesticides and contaminants in the collected grape vine canes. The removal of the outer bark can strongly reduce the risk of contamination, as pesticides are applied as a foliar treatment to the surface of the plant. Additionally, both sampling procedures (PH and PP) were performed after grape harvesting. In Europe, the use of pesticides and herbicides in grape cultivation is regulated by Regulation (EC) No. 1107/2009 of the European Parliament and of the Council of 21 October 2009 [59] concerning the placement of plant protection products. The regulation mandates adherence to safety intervals between the last application of pesticides and fruit harvest. Thus, we can assert that the collection of vine canes after grape harvest ensures greater phytosanitary safety and assumes the absence or presence of trace amounts of plant-protection products. Currently, there are no laws governing the maximum permissible content of plant-protection products in vine pruning. Nonetheless, Dorosh et al. [60] conducted a study that revealed pesticide levels in some samples of vine canes to be below those established for grapes.

### 2.4. HS-SPME-GC-MS Analysis

**Figure 6:**
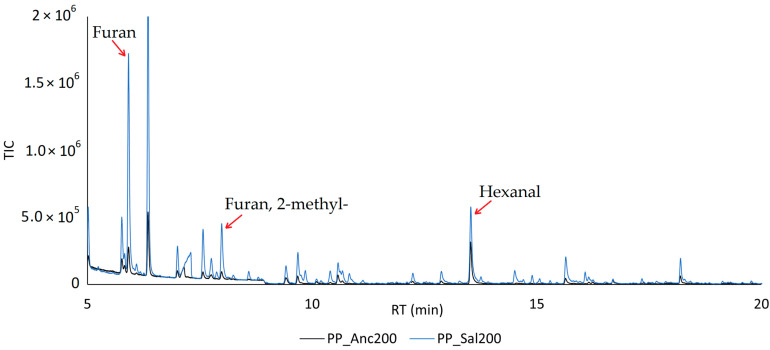
Overlay of chromatograms obtained by HS-SPME-GC-MS from PP_Anc200 and PP_Sal200 samples.

Figure 6 shows the HS-SPME chromatograms obtained from PP_Anc200 and PP_Sal200 samples processed with GC-MS instrumentation immediately after roasting. A combination of three criteria (i.e., mass spectra from the libraries supplied with the GC-MS software and databases, mass spectra found in the literature, and mass spectra and retention time of an injected standards) were used to identify the volatile compounds listed in Table 5. The reproducibility of the results is expressed as the standard deviation (SD) of three replicates.

Significant improvement in the quality of vinegars and alcoholic beverages aged in wood barrels is achieved by the strong increase in volatile compounds arising from the thermal degradation of the wood through the toasting process [61,62,63,64,65]. The chemical composition of the wood is significantly altered because of the pyrolysis and thermolysis reactions that occur during the toasting process. This is mainly due to the breakdown of the major biopolymers of wood such as lignin, polyosides, and lipids. This is reflected in the mass losses shown in Table 2 (Section 2.2), which indicate the extent of these transformations.

**Figure 7:**
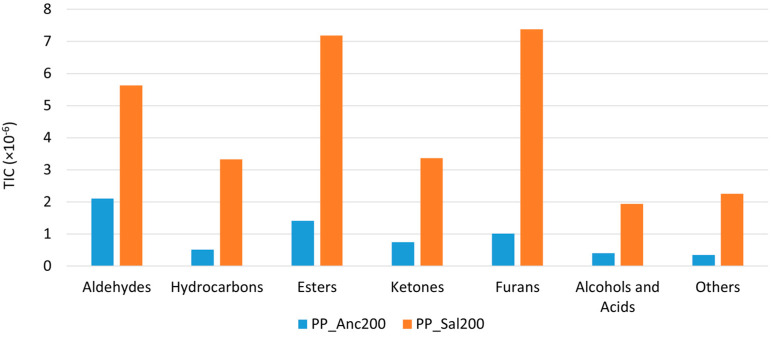
Results obtained by HS-SPME-GC-MS from PP_Anc200 and PP_Sal200 samples divided by chemical class.

The GC-MS analysis of PP_Anc200 and PP_Sal200 roasted minichips revealed the presence of 29 and 46 compounds, respectively. Figure 7 shows the sums of the TIC areas of the identified analytes, divided by chemical classes.

The PP_Sal200 sample showed a higher number of analytes across all molecular classes, as well as a larger total ion current (TIC) area for each. This observation is consistent with the findings reported in Table 2 (Section 2.2) concerning mass loss, where the Salamino samples showed a more marked release of VOCs following heat treatment. Therefore, we can affirm that the aroma of toasted cane shavings from Salamino grape variety has more intense notes. Ancellotta vine cane is probably more resistant to heat treatment, and a higher toasting temperature would be required to release a greater number of volatile aromas. Lignin degradation is reported to release phenolic compounds, such as guaiacol and syringol [61,62,65], completely absent in our samples, probably because lignin degradation typically occurs at higher roasting temperatures (>300 °C).

Many of the analytes detected are a result of processes caused by heat, such as the Maillard reaction, the breakdown of carbohydrates and sugars, changes in protein structure, and the oxidation of lipids [66,67,68]. The Maillard reaction is particularly important, as it gives many heat-processed foods their distinctive flavors and aromatic bouquets. The breakdown of amino acids through the Strecker process is a crucial step in the formation of key aroma compounds in the Maillard reaction, namely the Strecker aldehydes [4,66,67]. These species, which are 2-methyl propanal, 3-methyl butanal and 2-methyl butanal, are particularly powerful flavor contributors and are formed via the breakdown of valine, isoleucine, and leucine [69,70] or potentially via the lipid oxidation of unsaturated fatty acids [67,71]. These compounds are responsible for the rich, malty, and chocolate-like flavors of many foods [67].

Aldehydes accounted for 32% and 18% of the total headspace composition of PP_Anc200 and PP_Sal200, respectively. As a volatile compound, acetaldehyde is widely known for its fruity notes in many foods and beverages. It can be formed by various macromolecules, including α-alanine through Strecker degradation [66]. Hexanal, the most prevalent aldehyde, contributes to the green bean and cut-grass aroma, as well as the leafy and less ripe notes [4,10,66]. Linoleic acid degradation is one of the main sources of n-hexanal [69,72,73]. Another aldehyde, pentanal, is also produced during lipid oxidation reactions [69], and it contributes to the fresh and green notes of the samples, together with hexanal.

Ketones are commonly recognized as lipid oxidation by-products [72,74]. However, their formation is also linked to Strecker degradation and Maillard reactions [66].

Maillard-type reactions are a primary source of furan derivatives [75,76], although they are also generated through various other pathways [69,73], including the thermal oxidative degradation of polyunsaturated fatty acids, the thermal degradation of certain amino acids (such as serine, threonine, and cysteine), the breakdown of nucleosides, and the thermal degradation of carbohydrates, ascorbic acids, and other organic acids. Six furan derivatives were detected in our samples: furan; furan, 2-methyl-; furan, 2-ethyl-; furan, 2,5-dimethyl-; furan, 2,4-dimethyl-; and furfural. These accounted for 15% and 24% of the total headspace composition of PP_Anc200 and PP_Sal200, respectively. Sugar dehydration or fragmentation during the Maillard reaction possibly results in furfural and 2-methylfuran formation [69,72,75]. Moreover, 2-methylfuran is linked with specific amino acids, such as alanine and threonine [75]. Its formation process occurs via the combination of the corresponding Strecker aldehydes along with particular amino acids. Furan is found in a higher concentration in foods that are heat-treated. It is associated with several pathways, including amino acid degradation [77].

The only organic acid identified in our samples was acetic acid, which is known to be produced during Maillard reaction’s intermediate stage involving sugar moieties breaking down and dehydrating [66].

Four esters were detected in PP_Anc200 and PP_Sal200 samples. The formation of these compounds is linked to the breakdown of polyunsaturated fatty acids through lipid oxidation [66]. Short-chain esters are among the most significant aroma compounds in a wide range of food samples, such as cheese, wine, and apples [66,74,78,79]. Their contribution to the aroma is typically perceived as valuable, but if their concentration is excessive, they can generate unwanted fruit or fermented strong notes. Esters typically bring fruity notes with a sensory profile that can include hints of solvent, banana and pear, rose and honey, or apple and sweetness. These are key flavors compounds in several products derived from the grape plant or the winemaking processes [80], including its grape seeds [4].

The toasting process impacts the transformation of wood lipids into lactones, which play an important role in the final flavor and aroma. One of these compounds was identified only in the PP_Sal200 sample, namely 3(2H)-Furanone, dihydro-2-methyl-, which has been found in many studies on the brown sugar aroma, since it is associated with the Maillard reaction [81]. It imparts caramel and sweet notes.

We can therefore state that in the PP_Anc200 sample, the green notes relating to the aldehydes prevail, while the aromatic profile of the PP_Sal200 sample is more complex, as it characterized by a greater number of analytes relating to Maillard degradation, such as furan derivatives.

## 3. Materials and Methods

### 3.1. Reagents and Standards

Butanal, 3-methyl-; acetic acid, methyl ester-; ethyl ester; acetone were obtained from Sigma-Aldrich products, distributed by Merck KGaA, Darmstadt, Germany. Ethanol (96%); furfural; acetic acid; 2-butanone; hexanal; 1-decanol; n-hexane; nonane; dodecane; tetradecane; and hexadecane were obtained from Carlo Erba Reagents, Milano (Italy). HPLC-grade methanol and formic acid were obtained from Sigma Aldrich (St. Luis, MO, USA). Deionized water is produced by a Milli-Q Plus (Millipore system). *Trans*-resveratrol standard was obtained from Sigma Aldrich, distributed by Merck KGaA, Darmstadt, Germany.

### 3.2. Samples Preparation

The woody stems of *Vitis vinifera* cv. Ancellotta (Anc) and Salamino (Sal) were collected in a farm of the territorial district of Modena (Italy). Therefore, all were cultivated under the same conditions of soil, climate, and hydric regime. For the analytical procedures, we selected only the internal parts of the vine canes to avoid any contamination by pesticides and fungicides, which, if present, were superficially deposited on the bark. Therefore, the outer layer was peeled off and manually removed.

One sampling was carried out in September 2022, and the pruned canes were air-dried at room temperature for one week (post-harvest samples: PH_Anc; PH_Sal). A second sampling was conducted during annual pruning activities in February 2023, when the grapevine canes were partially and naturally dry on the plant (post-pruning samples: PP_Anc; PP_Sal). To limit the intrinsic variability of the plant material, the second sampling was conducted on the same plants that had generated the PH samples 6 months before.

**Figure 8:**
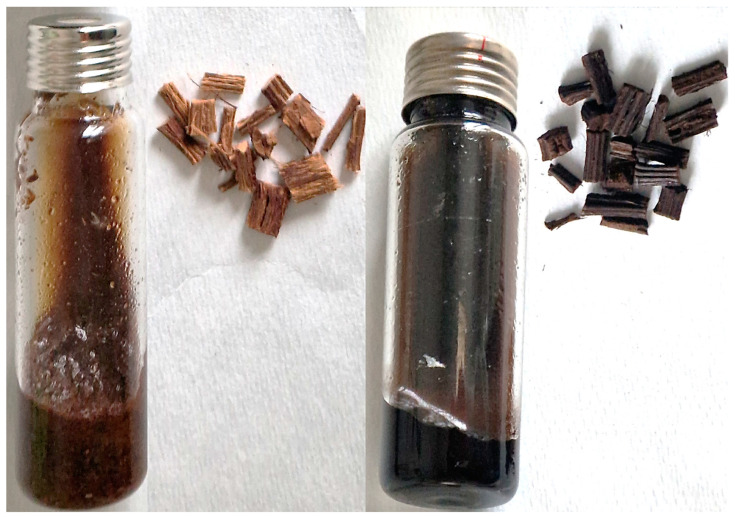
PP_Anc200 (sx) and PP_Anc240 (dx) minichips and extracts during maceration.

All the samples were manually dehulled, minced, reduced to 4–5 mm minichips, closed in glass containers, and then thermally treated for 2 h at different temperatures: 180 °C, 200 °C, 220 °C, and 240 °C in inert atmosphere (N_2_). They were then manually ground into powder and extracted through maceration with ethanol (Figure 8).

### 3.3. Proximate Composition

Moisture, ash, elemental analysis, and crude protein content were determined following the methods recommended by the Association of Official Analytical Chemists [82]. Moisture content was determined by drying the samples at 105 °C to a constant weight. The ash content was determined using a laboratory furnace at 550 °C and the temperature was gradually increased. The Dumas method was used to determine nitrogen content, which was converted to protein content multiplying by 6.25 factor [4,83]. Each measurement was performed in triplicate and the results were averaged.

### 3.4. Macerative Solvent Extraction

For the extraction of the active components, 2 g (±1%) of milled vine-shoots was placed into the extraction vessel, covered with ethanol 96% *v*/*v* (10 mL/g of powdered material) and closed with PBT screw cap. They were sonicated for 1 h at 35 °C and macerated for 24 h in an oven at 80 °C. The extracts were then filtered and the solid was washed three times with 10 mL of ethanol to collect the washings in the filtrate. The extracts were dried using a rotary evaporator and dried in an oven at 70 °C for 2 h before quantification. The final products had a pitchy/powdery consistency and were stored at 4 °C until analysis.

### 3.5. HPLC-DAD Analysis

The quantification of stilbenes was performed using HPLC-DAD analysis. *Trans*-resveratrol standards were processed and analyzed in replicates. Analytes were separated using a C18 Cortecs column (2.7 μm, 2.1 mm × 100 mm) (Waters Co., Milford, MA, USA). The analysis was performed at a constant flow rate of 0.3 mL/min, using a binary gradient elution with acidified water (0.1% formic acid) and methanol 80:20 (mobile phase A) and methanol 100% (mobile phase B). The gradient was programmed as follows: 0 min, 20% B; 25 min, 98% B, and held for 5 min to return to the initial conditions. Finally, a conditioning cycle of 10 min under the initial conditions was adopted. UV detection was performed at 280 nm and 325 nm. The stilbenoid content was determined from the calibration curve of the resveratrol standards (injected concentrations ranging from 2 to 500 μg/mL). The linearity of the response (correlation coefficient *R^2^* = 0.9891) of the *trans*-resveratrol standards was determined by plotting the peak area versus its concentration.

### 3.6. UHPLC-MS Analysis

A UHPLC system coupled with an Orbitrap Q-Exactive equipped with a micro-ESI (Thermo Fisher Scientific, Waltham, MA, USA) was used. All the samples were processed and analyzed in replicates. Analytes were separated using a C18 Cortecs column (2.7 μm, 2.1 mm × 100 mm) (Waters Co., Milford, MA, USA). The analysis was performed under the same conditions as those used for the quantification of *trans*-resveratrol via HPLC-DAD analysis. The analysis was performed at a constant flow rate of 0.3 mL/min, using a binary gradient elution with acidified water (0.1% formic acid) and methanol 80:20 (mobile phase A) and methanol 100% (mobile phase B). The gradient was programmed as follows: 0 min, 20% B; 25 min, 98% B held for 5 min to then return to the initial conditions. Finally, a conditioning cycle of 10 min with the initial conditions was adopted. Electrospray ionization in negative mode was used. Detection was performed considering a mass range of 50–1200 u.m.a.

Compounds in the extracts were identified according to their mass spectrum and, for *trans*-resveratrol only, the retention time was compared to the external standard. Quantification was performed using an external calibration curve with *trans*-resveratrol as the external standard, and the results were expressed as *trans*-resveratrol equivalents.

### 3.7. HS-SPME-GC-MS

About 2 g of PP_Anc200 and PP_Sal200 shives samples were placed in a 7 mL vial. Extraction was performed in the sample headspace, maintained at 40 °C for 15 min, using a DVB/CAR/PDMS fiber and 50/30 μm film thickness (Supelco, Bellefonte, PA, USA) housed in its manual holder (Supelco Inc., Bellefonte, PA, USA). The SPME fiber was then introduced into the GC-MS splitless injector (250 °C) and the thermal desorption time was 15 min. The experimental procedure was performed on three replicate samples interspersed with a SPME blank analysis. No artifacts were observed in the SPME blank analysis.

An Agilent 6890N Network as a chromatography system coupled with a 5973N mass spectrometer (Agilent Technologies, Santa Clara, CA, USA) was used for GC-MS analysis. Chromatographic separation was performed using a DB-5MS UI column (60 m × 0.25 mm i.d., 1.00 μm film thickness; J&W Scientific, Folsom, CA, USA). Helium (He) as the carrier gas was maintained at a constant flow rate of 1 mL/min, and the column head pressure was 15 psi. The initial oven temperature was maintained for 5 min at 40 °C, followed by a heating ramp at 8 °C/min up to 160 °C and then at 10 °C/min to reach a final temperature of 270 °C, held for 5 min. The transfer line was maintained at 270 °C. The electron impact (EI) at 70 eV was the ionization mode of the mass spectrometer, and the full scan acquisition mode was selected, with a *m*/*z* scan range from 25 to 300. Enhanced ChemStation software (Agilent Technologies, Santa Clara, CA, USA) was used to analyze chromatograms and mass spectra. The tentative identification of the VOCs was achieved by comparing the mass spectra with the data system library (NIST14/NIST05/WILEY275/NBS75K) and by using web databases, such as the National Institute for Standards and Technology (NIST database https://webbook.nist.gov, accessed on 25 November 2022) and Mass Bank of North America (https://mona.fiehnlab.ucdavis.edu, accessed on 26 November 2022). The linear retention index (LRI) was used to compare our data with those reported in the literature and in the NIST Standard Reference Database. The LRI values were calculated from a solution of n-alkanes (C6, C9, C12, C14, and C16) analyzed following the same procedure as that used for the samples. The injection of pure standards, analyzed under the same operating conditions of the samples, was used for the identification of some analytes. The amount of each identified VOC is expressed as the total ion current (TIC) peak area. The results are expressed as mean ± standard deviation (SD) of at least three replicates.

### 3.8. Statistical Analysis

Statistical analysis was performed using XLstat (Addinsoft, Paris, France) [84]. Principal component analysis (PCA) was performed using Pearson’s correlation to investigate stilbenoid profiles and sample relationships.

## 4. Conclusions

The potential of roasted canes from two Lambrusco grapevine cultivars typical of the Modena and Reggio Emilia district was evaluated using HPLC-MS and HS-SPME-GC-MS. These canes can be effectively used in the production of vinegars and alcoholic beverages as shavings infused during aging.

Generally, increasing the roasting temperature in the 180–240 °C range produced the maximum extraction capacity at 220 °C. However, a parallel progressive decrease in the total stilbenoid content was observed, probably because of their thermolability, regardless of the grape cultivar or sampling time. Interestingly, the Ancellotta samples exhibited greater heat resistance and stilbenoids concentration than the Salamino samples. Finally, we observed that upon heat treatment, Salamino cultivar samples release a greater number of volatile aromas, resulting in a more complex aromatic profile with a higher number of Maillard reaction-related compounds, such as furan derivatives.

## Figures and Tables

**Table 1:** Proximate chemical composition of the pruning cane samples.

	PH_Anc	PP_Anc	PH_Sal	PP_Sal
Moisture (at 105 °C)	15.87 ± 0.32	13.26 ± 0.24	23.52 ± 0.23	18.12 ± 0.25
C% *	39.64 ± 0.22	46.56 ± 0.36	37.32 ± 0.30	45.31± 0.40
H% *	6.51 ± 0.08	9.15 ± 0.09	3.65 ± 0.05	5.72 ± 0.10
N% *	0.46 ± 0.03	0.57 ± 0.05	0.38 ± 0.06	0.47 ± 0.04
S% *	<0.1	<0.1	<0.1	<0.1
O% *^#^	50.32 ± 0.29	40.49 ± 0.56	55.84 ± 0.39	45.44 ± 0.21
Ash% *	2.84 ± 0.05	3.23 ± 0.06	2.81 ± 0.08	3.06 ± 0.05
Protein content% *	2.87 ± 0.34	3.56 ± 0.31	2.37 ± 0.5	2.93 ± 0.25

* on a dry basis. ^#^ by difference. All values are expressed as mean ± standard deviation of three replicates.

**Table 2:** Mass loss following roasting of the grapevine cane samples.

Roasting t/°C	PH_Anc	PP_Anc	PH_Sal	PP_Sal
180	45.74 ± 0.43	36.81 ± 0.42	55.75 ± 0.41	46.55 ± 0.38
200	46.62 ± 0.40	37.68 ± 0.41	56.62 ± 0.39	47.83 ± 0.34
220	49.21 ± 0.44	40.42 ± 0.42	59.14 ± 0.40	50.96 ± 0.54
240	55.98 ± 0.41	47.13 ± 0.43	65.84 ± 0.37	58.01 ± 0.51

All values are expressed as mean ± standard deviation of three replicates.

**Table 3:** Extraction yield of roasted vine shoot samples.

	Extraction Yield% *			
Roasting t/°C	PH_Anc	PP_Anc	PH_Sal	PP_Sal
180	1.97 ± 0.09	2.22 ± 0.10	2.19 ± 0.08	2.47 ± 0.15
200	2.03 ± 0.10	2.35 ± 0.09	2.30 ± 0.11	2.56 ± 0.13
220	2.15 ± 0.12	2.44 ± 0.09	2.42 ± 0.07	2.72 ± 0.11
240	1.84 ± 0.09	2.06 ± 0.08	2.01 ± 0.09	2.45 ± 0.12

* on the dry basis of the extract, determined against the roasted sample. All values are expressed as mean ± standard deviation of three replicates.

**Table 4:** Stilbenoid concentrations in grape pruning cane samples roasted at different temperatures.

	Stilbenoids Concentration (mg/kg) ^1,2^				
Sample	*trans*-Resveratrol	*trans*-Piceatannol	*trans*-ε-Viniferin	Main Trimer	Total
PH_Anc180	815.9 ± 153	153.4 ± 46.0	261.4 ± 60.9	93.0 ± 23.4	1323 ± 283
PP_Anc180	2176 ± 366	339.8 ± 111	555.4 ± 114	152.2 ± 46.9	3223 ± 638
PH_Sal180	706.4 ± 230	139.5 ± 61.6	224.1 ± 25.2	75.4 ± 14.8	1145 ± 332
PP_Sal180	1786 ± 301	304.1 ± 101	489.8 ± 70.2	140.2 ± 29.8	2720 ± 502
PH_Anc200	693.1 ± 134.6	139.0 ± 48.2	241.0 ± 34.7	59.4 ± 11.7	1132 ± 229
PP_Anc200	2044 ± 427	275.5 ± 94.0	520.6 ± 80.0	135.7 ± 24.0	2976 ± 625
PH_Sal200	508.8 ± 188.3	116.3 ± 38.5	201.4 ± 50.8	43.6 ± 8.5	870.2 ± 286.1
PP_Sal200	1463 ± 277	346.4 ± 76.1	491.1 ± 69.9	129.2 ± 56.6	2330 ± 480
PH_Anc220	548.8 ± 114.5	102.2 ± 27.5	180.4 ± 20.1	NQ	831.5 ± 162.1
PP_Anc220	1671 ± 255	226.5 ± 40.5	439.9 ± 59.9	97.1 ± 22.9	2435 ± 378
PH_Sal220	381.3 ± 83.9	93.76 ± 20.3	167.7 ± 30.5	NQ	642.8 ± 134.7
PP_Sal220	1203 ± 112	201.8 ± 41.0	428.7 ± 68.8	83.7 ± 16.3	1917 ± 238
PH_Anc240	195.8 ± 82.3	NQ	145.5 ± 45.0	NQ	341.3 ± 127.3
PP_Anc240	831.3 ± 181.4	188.8 ± 42.9	359.3 ± 59.9	NQ	1379 ± 284
PH_Sal240	105.9 ± 33.7	NQ	133.3 ± 31.8	NQ	239.3 ± 65.5
PP_Sal240	667.8 ± 37.7	128.3 ± 28.2	326.9 ± 56.5	NQ	1123 ± 122

^1^ Data are expressed as mean ± standard deviation of three replicates. ^2^ Data are expressed as *trans*-resveratrol equivalents [40]. NQ means detected but not quantified because of very low levels.

**Table 5:** VOC composition of PP_Anc200 and PP_Sal200 samples, identified through HS-SPME-GC-MS analysis, grouped by chemical classes. Data are expressed as mean (*n* = 3), TIC area × 10^−6^ ± SD.

Compound	LRI	ID ^#^	Aroma	PP_Anc200	PP_Sal200
				Area × 10^−6^	Area × 10^−6^
**Aldehydes**					
Acetaldehyde	430	A, B	Pungent, fresh, lifting, fruity, musty	1.75 ± 0.07	5.51 ± 0.12
Propanal	470	A, B	Pungent, earthy, wine, nutty, cocoa	1.53 ± 0.06	3.76 ± 0.09
Propanal, 2-methyl-	512	A, B	Fresh, aldehydic, floral, green	1.39 ± 0.09	6.25 ± 0.11
Butanal	592	A, B	Pungent, cocoa, musty, green, malty	-	0.186 ^a^
Butanal, 3-methyl-	597	A, B, C	Aldehydic, chocolate, peach, fatty	1.82 ± 0.06	4.65 ± 0.07
Butanal, 2-methyl-	606	A, B	Musty, cocoa, coffee, nutty, malty	1.70 ± 0.06	7.17 ± 0.19
Pentanal	638	A, B	Fermented, bready, fruity, berry	2.12 ^a^	4.70 ± 0.12
Hexanal	742	A, B, C	Green, fatty, leafy, vegetative, fruity, clean	10.3 ± 0.1	22.1 ± 0.3
Heptanal	834	A, B	Fresh, aldehydic, fatty, green, herbal	0.442 ^a^	2.02 ^a^
**Organic acids and alcohols**					
Acetic acid	530	A, B, C	Sharp, pungent, sour, vinegar	3.80 ± 0.15	18.0 ± 0.3
1-Penten-3-ol	621	A, B	Ethereal, green, radish, vegetable, fruity	-	1.14 ^a^
1-Pentanol	705	A, B	Pungent, fermented, bready, yeasty	0.260 ^a^	0.324 ± 0.082
**Esters**					
Formic acid, methyl ester	442	A, B	Fruity, plum, ester	1.75 ± 0.08	10.4 ± 0.09
Formic acid, ethyl ester	483	A, B, C	Fruity	-	0.902 ^a^
Acetic acid, methyl ester	488	A, B, C	Ethereal, sweet, fruity, winey	11.7 ± 0.08	56.6 ± 0.21
Propanoic acid, methyl ester	567	A, B	Fresh, harsh, rum, fruity	0.705 ^a^	3.96 ± 0.18
**Ketones**					
Acetone	468	A, B, C	Solvent, ethereal, apple, pear	2.51 ± 0.14	11.2 ± 0.4
2,3-Butanedione	532	A, B	Butter, sweet, creamy, pungent, caramel	2.39 ± 0.08	12.1 ± 0.2
2-Butanone	538	A, B, C	Acetone, ethereal, fruity, camphor	2.14 ± 0.12	6.18 ± 0.09
2,3-Pentanedione	632	A, B	Pungent, sweet, butter, creamy, nutty	0.417 ± 0.071	3.86 ± 0.06
3-Pentanone, 2-methyl-	692	A, B	-	-	0.354 ± 0.092
**Furan derivatives**					
Furan	473	A, B	Ethereal	5.51 ± 0.13	46.4± 0.4
Furan, 2-methyl-	546	A, B	Ethereal, acetone, chocolate	3.26 ± 0.17	14.7 ± 0.3
Furan, 2-ethyl-	641	A, B	Beany, cocoa, bread, malty, coffee	0.931 ± 0.105	3.63 ± 0.23
Furan, 2,5-dimethyl-	647	A, B	Chemical, ethereal, meaty, gravy, roast	0.182 ^a^	3.60 ± 0.11
Furan, 2,4-dimethyl-	657	A, B	-	-	1.23 ± 0.08
Furfural	776	A, B, C	Sweet, woody, bready, caramel, phenolic	0.298 ± 0.079	4.22 ± 0.09
**Hydrocarbons**					
1-Propene, 2-methyl-	432	A, B	-	3.72 ^a^	13.4 ± 0.12
Butane	435	A, B	-	-	3.85 ± 0.09
1-Butene	438	A, B	-	-	1.43 ± 0.08
1,3-Butadiene, 2-methyl-	479	A, B	-	0.380 ^a^	2.74 ± 0.11
2-Butene, 2-methyl-	486	A, B	-	-	0.608 ± 0.084
Hexane	542	A, B	-	-	2.14 ± 0.09
Cyclopentane, methyl-	575	A, B	-	-	1.57 ± 0.12
1,3-Pentadiene, 3-methyl-	578	A, B	-	-	1.56 ± 0.08
4-Methyl-1,3-pentadiene	581	A, B	-	-	0.239 ^a^
Cyclohexane	612	A, B	-	-	3.41 ± 0.06
Nonane	831	A, B	-	-	2.38 ± 0.10
Cycloheptene	853	A, B	-	1.09 ± 0.06	-
**Others**					
Benzene	608	A, B	Aromatic	-	1.98 ^a^
1H-Pyrrole, 1-methyl-	684	A, B	Woody, smoky, herbal	0.138 ^a^	0.460 ± 0.081
Disulfide, dimethyl-	696	A, B	Sulfurous, vegetable	0.724 ± 0.095	3.13 ± 0.11
Toluene	719	A, B	Sweet	0.986 ± 0.077	4.20 ± 0.09
3(2H)-Furanone, dihydro-2-methyl-	750	A, B	Sweet, solvent, bread, buttery, nutty	-	2.27 ± 0.09
Pyridine, 2,5-dimethyl-	790	A, B	Roasted, green, earthy	-	1.88 ± 0.08
Ethylbenzene	809	A, B	-	-	0.387 ± 0.075
Xylene	816	A, B	-	1.62 ± 0.09	8.28 ± 0.10

^#^ The identification of the components was obtained via: (A) the mass spectral data of the libraries supplied with the operating system of the GC-MS and from mass spectra databases; (B) the mass spectra found in the literature; and (C) the mass spectra and retention time of an injected standard. ^a^ SD < 0.05.

## Data Availability

Data are contained within the article.
